# Associations between eating jetlag and adiposity in preschool children, and the moderating roles of social jetlag and chronotype

**DOI:** 10.1007/s00394-026-04015-6

**Published:** 2026-06-29

**Authors:** Mirkka Maukonen, Anna M. Ruokolahti, Josefine Björkqvist, Henna Launistola, Jenna Rahkola, Ilse Tillman, Henna Vepsäläinen, Carola Ray, Eva Roos, Maijaliisa Erkkola, Reetta Lehto

**Affiliations:** 1 Folkhälsan Research Center, Helsinki, Finland; 2 Finnish Institute for Health and Welfare, Helsinki, Finland; 3 University of Tampere, Helsinki, Finland; 4 University of Helsinki, Helsinki, Finland; 5 University of Aberdeen, Aberdeen, UK; 6 Uppsala University, Uppsala, Sweden

**Keywords:** BMI, Chrononutrition, Chronotype, Circadian rhythms, Eating time variation, Obesity, Waist-to-height ratio

## Abstract

**Purpose:**

Eating jetlag (variation in eating times between weekdays and weekends) may increase obesity risk in adults, but studies in children are lacking. The potential moderating roles of social jetlag (SJL, variation in sleep timing between weekdays and weekends) and chronotype (an individual’s intrinsic circadian rhythm) also remain unclear. We examined whether eating jetlag is associated with adiposity in Finnish preschoolers and whether chronotype or SJL moderate these associations.

**Methods:**

Data included 639 children (3–6 years, 48% girls) from the cross-sectional DAGIS study (2015–2016). Diet was assessed using 3-day food records. SJL and chronotype were assessed from hip-worn accelerometer data. Six eating jetlag indicators were derived as weekend–weekday differences: first/last eating occasion jetlag, eating/energy intake midpoint jetlag and morning/evening latency jetlag. Adiposity was measured by BMI z-score and waist-to-height ratio (WHtR). Linear regression with interaction terms was used, with significant interactions examined in moderator-stratified analyses.

**Results:**

Eating jetlag indicators were not associated with adiposity in the full sample. However, interaction analyses indicated moderation by SJL. Among children with high SJL (median 62 min, IQR 17), greater eating midpoint jetlag was associated with lower BMI z-score (β: − 0.65, 95% CI: − 1.21 to − 0.10), whereas greater morning latency jetlag was associated with higher BMI z-score (0.83, 0.19 to 1.46) and WHtR (0.035, 0.013 to 0.058). No consistent interactions were observed for chronotype.

**Conclusion:**

Although no associations were observed between eating jetlag and adiposity in the full sample, associations varied by SJL. However, these findings require confirmation in longitudinal studies.

**Supplementary Information:**

The online version contains supplementary material available at 10.1007/s00394-026-04015-6.

## Introduction

Emerging evidence suggests that not only *what* we eat, but also *when* we eat, may play a role in health [1]. This concept is captured by the term chrononutrition, which encompasses the timing, frequency, and regularity of eating. The circadian system, regulated by central and peripheral clocks, plays an important role in coordinating metabolism [1, 2]. The central clock in the brain synchronizes peripheral clocks across the body, including those in metabolic organs, via hormonal and neural pathways. While light is the primary synchronizer of the central clock, food intake is considered as one of the key synchronizers for peripheral clocks and eating at inconsistent times can disrupt the alignment between these clocks and impair metabolic balance [3, 4]. Consequently, previous studies in children and adults have linked breakfast skipping and late eating, particularly in relation to sleep timing, to higher adiposity [5–8]. In line with these findings, eating close to sleep onset was associated with a higher waist-to-height ratio (WHtR) also in our study population of preschool-aged children [9].

A less studied marker of eating timing irregularity is eating jetlag, which refers to the difference in eating timing between weekdays and weekends [10]. It can be quantified using several indicators, including the difference in timing of the first eating occasion (first EO jetlag) or last eating occasion (last EO jetlag), or, most commonly, the midpoint between them (eating midpoint jetlag). Eating jetlag resembles social jetlag (SJL), which refers to differences in sleep timing between weekdays and weekends and is commonly used as an indicator of circadian misalignment [11]. Another closely related concept is chronotype, which reflects an individual’s intrinsic circadian preference for sleep–wake timing (morningness–eveningness) [12]. Although these constructs are conceptually interrelated, as they are linked to sleep and behavioral timing, they capture different aspects of circadian behavior: chronotype reflects an individual’s intrinsic preference for sleep–wake timing, SJLreflects misalignment between biological and social timing, and eating jetlag reflects variability in the timing of food intake. SJL and chronotype are therefore important factors to consider, as they represent related but distinct dimensions of circadian variability that may influence how eating jetlag relates to adiposity.

Thus far eating jetlag indicators and their associations with adiposity have been studied only in adults. Among Spanish and Mexican students (*n* = 1121), eating midpoint jetlag was positively associated with BMI, independently of SJL and chronotype [10]. In Colombian women (*n* = 115), greater jetlag in first EO, nightly fasting duration, and energy intake after 8 pm were associated with higher BMI and waist circumference [13]. A recent study using NHANES data (*n* = 1184) suggested that chronotype may modify these associations, as they found that greater eating midpoint jetlag was associated with higher BMI only among adults with an evening chronotype [14]. However, the potential moderating role of SJL has not been examined in previous studies. Given that SJL reflects a risk for circadian misalignment [11] and has been linked to both eating timing and eating jetlag [10, 15, 16], as well as to adiposity-related outcomes [17–20], it is plausible that SJL may also modify how eating jetlag associates with adiposity.

Despite growing evidence in adults, the associations between eating jetlag and adiposity have not been studied in children. This gap in knowledge is critical, as childhood weight strongly predicts adult adiposity, highlighting the importance to identify early behavioral factors related to weight development for effective prevention [21]. Furthermore, parental practices largely shape children’s dietary habits in early childhood, and these early habits can track into adulthood [22, 23]. Studying eating jetlag in relation to adiposity at this age enables us to examine whether the associations seen in adults are already evident at an earlier age, despite children’s limited autonomy over their schedules. Thus, the aim of this study was to cross-sectionally examine whether different eating jetlag indicators are associated with adiposity (BMI z-score and WHtR) in Finnish preschool-aged children, and whether SJL or chronotype moderate these associations. Based on earlier literature [10, 13, 14], we hypothesized that greater eating jetlag would be associated with higher adiposity, and that these associations would be more pronounced among children with an evening chronotype. Due to the lacking prior evidence, the moderating role of SJL was examined exploratively in this context.

## Methods

### Study population

The study population comprised children aged 3 to 6 years attending Early Childhood Education and Care (ECEC) centers (hereafter referred to as preschools) from the DAGIS (Increased Health and Wellbeing in Children, Families and Educational Settings) Survey, conducted in 66 preschools in Southern and Western Finland between September 2015 and April 2016 [24]. The overall aim of the study was to examine energy balance-related behaviors and self-regulation skills among children. The study included assessments of children’s dietary habits (3-day food records), sleep (7 days, hip-worn accelerometers), and anthropometry, collected as part of a broader set of assessments. Parents or legal guardians provided informed consent for themselves and their children. A total of 864 children (24% of those invited) participated in the study. Of these, 639 children (74%) with complete data on 3-day food records, sleep (at least three valid weekday nights and one weekend night), and adiposity measures were included in the current study. The study was approved by the University of Helsinki Ethical Review Board in the Humanities and Social and Behavioral Sciences (6/2015).

## Outcome variables—anthropometric measurements

During preschool visits trained researchers measured children’s body weight, height and waist circumference. Weight was measured with portable bench scales (CAS PB-100/200) to the nearest 0.01 kg, and height with stadiometers (SECA 217) to the nearest 0.1 cm. Waist circumference was measured twice using SECA 201 measuring tapes at the midpoint between the iliac crest and the lower margin of the last palpable rib, and the mean of these two measurements was calculated.

BMI was calculated as weight (kg)/height (m²). BMI z-scores were computed based on Finnish age- and sex-specific growth references [25]. WHtR was derived by dividing the mean waist circumference (cm) by height (cm).

## Predictors—eating jetlag indicators

Eating jetlag indicators were derived from 3-day food records that were completed both at home and in preschool settings [26]. Parents recorded all foods and beverages consumed outside preschool on two assigned weekdays and one weekend day ensuring that all days of the week were represented across participants. Each entry included the time, location, and portion sizes of foods, with portion sizes estimated using a validated Children’s Food Picture Book [27] or household measurement tools. In preschools, preschool staff recorded children’s food intake on the same weekdays that the families kept a food record at home using pre-coded two-day food records, applying the same portion size estimation methods. All records were reviewed by trained research assistants and missing or unclear information was clarified when necessary.

Dietary data were analyzed using AivoDiet software (version 2.2.0.0, Mashie FoodTech Solutions Finland Oy) and the Fineli Food Composition Database (Release 16, 2013) administered by the Finnish Institute for Health and Welfare.

Six eating jetlag indicators were derived (Table 1), all of which reflected differences in eating timing patterns between weekdays and weekends. The indicators were calculated by subtracting weekend values from weekday values. All analyses were conducted using the absolute values of these differences to reflect the magnitude of variation in eating timing between weekends and weekdays. This approach aligns with previous studies to ensure methodological comparability [10, 13, 14].

Unlike other eating jetlag indicators, the calculation for morning and evening latency jetlag (Table 1) required information on sleep timing in addition to food record data. Sleep times were derived from accelerometer data, as described in the following subsection. To calculate these indicators, matched data on both eating and sleep were required for at least one weekday and one weekend day. Based on the availability of matched days, morning latency jetlag was calculated for 552 children (64%) and evening latency jetlag for 618 children (72%). We excluded an additional 24 children from the morning latency jetlag analysis and 15 from the evening latency jetlag analysis due to negative latency values, which likely reflected timing inconsistencies or reporting errors (e.g., energy intake recorded before waking or after sleep onset). As latency is defined as eating occurring after waking and before sleep onset and therefore these values were excluded from the analyses. In total, 528 children (61%) were included in the final analytical sample for morning latency jetlag and 603 children (70%) for evening latency jetlag.

**Table 1:** Descriptions of eating jetlag indicators

Eating jetlag indicator	Definition	Formula
First EO^1^ jetlag	Absolute difference in the clock time of the first EO	|Weekend first EO– Weekday first EO|
Last EO^1^ jetlag	Absolute difference in the clock time of the last EO	|Weekend last EO – Weekday last EO|
Eating midpoint jetlag	Absolute difference in eating midpoint. Eating midpoint is the clock time halfway between the first and the last EO	|Weekend eating midpoint – Weekday eating midpoint|
Energy midpoint jetlag	Absolute difference in energy intake midpoint. Energy intake midpoint is the clock time at which half of the daily energy intake has been consumed	|Weekend energy midpoint – Weekday energy midpoint|
Morning latency jetlag	Absolute difference in morning latency. Morning latency is the time between sleep offset and the first EO	|Weekend morning latency – Weekday morning latency|
Evening latency jetlag	Absolute difference in evening latency. Evening latency is the time between the last EO and sleep onset	|Weekend evening latency– Weekday evening latency|

EO; eating occasion.

^1^An EO was defined as any separately reported intake of at least 104.6 kJ (25 kcal) within 15-min time period in accordance with our previous study [9] to account for portion sizes typically consumed by this age group.

## Potential moderators—social jetlag and chronotype

Sleep was assessed using hip-worn ActiGraph w-GT3X-BT accelerometers (ActiGraph, Pensacola, FL, USA), worn continuously for 24 h across 7 consecutive days and nights, except during water-based activities. Valid 24-hour periods were determined using the GGIR R package (v2.5), which was used for preprocessing, including autocalibration and non-wear time detection, and required a minimum of 16 h of wear time within a noon-to-noon window [28, 29]. Estimates for evening sleep onset and morning wake-up times were then derived using a data-driven, open-source Hidden Markov Model (HMM)-based algorithm (the “hmmacc” R package), which classifies sleep–wake states based on individual activity profiles without relying on sleep diaries, age-specific thresholds, or device-dependent settings [30]. Although this algorithm has originally been validated against polysomnography in adults [30], it has previously been applied and described in detail in the DAGIS study population showing good agreement between parent-reported sleep and accelerometer-derived estimates [29]. For the current study, children were required to have data from at least three valid weekday nights and one valid weekend night. A weekend night was defined as the night between Friday and Saturday or between Saturday and Sunday.

SJL and chronotype were derived from sleep onset and wake-up times. SJL was defined using the sleep-corrected approach (SJLsc), which adjusts for potential weekly sleep debt [31]. The SJLsc formula ultimately reduces to the following: if the free day (weekend) sleep onset was later and duration longer than on non-free days (weekdays), SJLsc was calculated as the absolute difference in sleep onset times. Otherwise, if weekday sleep duration was longer and sleep offset occurred earlier than on weekend days, SJLsc was calculated as the absolute difference in sleep offset times. For comparison, we also used the original formula for SJL (SJL₀), defined as the absolute difference between the midpoints of sleep (MSF; the halfway point between sleep onset and wake-up) on weekends and weekdays [11].

SJLsc was used as a continuous variable, where higher values indicated greater discrepancies in sleep timing between weekdays and weekends. For stratified analyses, SJLsc was categorized into three groups based on percentiles: lowest 10% (low), middle 80% (moderate), and highest 10% (high). As there are no standardized cut-offs for SJL, this explorative approach was chosen to capture sufficient variability in the current study population, especially at the extremes of distribution, which were of primary interest. Previous studies, mainly in adults [32], have often used hour-based thresholds, typically considering SJL from one hour upwards. Applying this cut-off in our data resulted in a substantially smaller high-SJLsc group and thus using percentile-based categorization ensured larger group sizes for stratified analyses, with children in the highest group having an average SJLsc exceeding one hour. A similar approach was used for SJLo stratifications.

Chronotype was estimated using the Munich ChronoType Questionnaire (MCTQ) approach and defined as the midpoint of sleep on free days (weekends), corrected for potential weekly sleep debt (MSFsc) [12, 33]. The correction was applied if weekend sleep duration exceeded average weekday sleep duration, using the formula:

MSFsc = MSF − (weekend sleep duration - average weekly sleep duration)/2.

In cases where weekend sleep duration did not exceed the average weekday sleep duration, and therefore no correction was needed, MSFsc was equal to MSF. MSFsc was treated as a continuous variable (clock time), where higher values indicated a later chronotype. In stratified analyses, chronotype was categorized into three groups (10%–80%–10%) based on previous reports on the prevalence of evening chronotypes in similarly aged children (10%–26%) [34–37] and in accordance with a previous study in the DAGIS population [38]. Children in the lowest 10th percentile were categorized as having a morning chronotype tendency, those in the highest 10th percentile as having an evening chronotype tendency, and those in between as having an intermediate chronotype tendency.

## Confounding factors

Potential confounding factors were selected based on previous literature [9, 10, 13, 14] and included the child’s age, sex, parental education level, moderate to vigorous physical activity (MVPA), sleep duration and total daily energy intake. Parents reported the child’s birth date and sex, and parental education level(s). The child’s age (years) was calculated by subtracting the birthday date from the research date. The highest familial parental education was divided into low (i.e. high school or vocational diploma or less), medium (i.e. associate or bachelor’s degree), and high (i.e. master’s, licentiate or doctoral degree) categories. Data for MVPA and for sleep duration was derived from accelerometer data. The amount of MVPA (min/hour) for this age group was determined using the Evenson cut points [39]. Sleep duration (hours) was defined as the as the time between sleep onset and wake-up. Total daily energy intake (kJ/day) was derived from the food records.

### Statistical analyses

The characteristics of the study participants are reported as means with standard deviations (SD) for continuous variables or medians with interquartile ranges (IQR) for continuous skewed variables and as proportions for categorical variables, stratified by the SJLsc groups. Differences between SJLsc groups were tested using analysis of variance (ANOVA) for continuous variables or the Kruskal–Wallis test for skewed continuous variables, followed by a Tukey post-hoc test or the Wilcoxon rank-sum test for skewed variables if the overall ANOVA/Kruskal–Wallis test was statistically significant. For categorical variables the chi-squared test was used to test differences between SJLsc groups.

Linear regression analysis was used to examine the overall associations between each eating jetlag indicator (Table 1) and the outcomes (BMI z-score and WHtR).

Two main models were used to adjust for potential confounders:

Model 1: Adjusted for children’s age and sex.

Model 2: Further adjusted for parental education level, MVPA, sleep duration, and total daily energy intake.

Two additional models were also tested in which SJLsc/SJLo and chronotype were separately included as confounders in Model 2. As these additions did not alter the results, they were omitted from the final models.

To assess potential moderation, we included interaction terms between SJLsc/SJLo or chronotype and each eating jetlag indicator in separate models. The interaction analyses were conducted using Model 2 adjustments, and the interacting variables were treated as continuous. Statistically significant interactions were further examined using moderator-stratified linear regression analyses.

The normality of residuals in the linear regression models was assessed visually using Q–Q plots along with other standard diagnostic plots (residuals vs. fitted, scale–location, and residuals vs. leverage). For WHtR, some deviation from normality was observed, and we therefore tested log-transformed models to address potential skewness. However, as the results remained unchanged, we report the untransformed models for better interpretation.

All analyses were conducted using R statistical software (version 4.4.2) [40]. A P-value < 0.05 was considered statistically significant in all analyses.

## Results

### Descriptives of the total study sample and by SJLsc groups

The mean age of the children was 4.8 years, and 48% were girls (Table 2). The median SJLsc was 20 min (26 min for SJLo), and the mean chronotype (MSFsc) was 2:17. Median values of eating jetlag indicators ranged from 25 min (last EO jetlag) to 50 min (first EO jetlag). Children in the high SJLsc group had a 60-minute higher median SJLsc and a 47-minute later chronotype compared to the low SJLsc group. They also had greater eating midpoint jetlag (48 vs. 20 min) and first EO jetlag (64 vs. 50 min).

Descriptives of the source variables for the eating jetlag indicators are presented in Supplemental Table 1. Eating midpoints, energy intake midpoints, first EO, and sleep timing (onset and wake-up) were generally later on weekends than on weekdays. These variables, along with the last EO, were also later in children with high SJLsc compared to those with low SJLsc, particularly on weekends (*P* < 0.05).

**Table 2:** Descriptives of characteristics of the total study sample and by social jetlag corrected for sleep debt (SJLsc) groups. Data are presented as mean (SD) or n (%) for categorical variables, unless otherwise indicated

	All	SJLsc			P-value^1^
		low	moderate	high	
	n = 639	n = 64 (10%)	n = 511 (80%)	n = 64 (10%)	
***Confounding factors***					
Age (years)	4.8 (0.9)	4.7 (1.0)	4.8 (0.9)	4.7 (0.9)	0.26
Sex (girls)	306 (48%)	28 (44%)	246 (49%)	32 (50%)	0.75
Parental education level^2^					0.54
low	132 (21%)	17 (27%)	99 (19%)	16 (25%)	
medium	271 (42%)	26 (41%)	217 (43%)	28 (44%)	
high	234 (37%)	21 (32%)	193 (38%)	20 (31%)	
MVPA (min/hour)	5.40 (1.60)	5.29 (1.52)	5.25 (1.33)	5.09 (1.49)	0.40
Sleep duration (hh:mm)	9.42 (0.31)	9.39 (0.34)	9.43 (0.31)	9.41 (0.35)	0.55
Energy intake (kJ/day)	5796 (1120)	5823 (1213)^b,c^	5836 (1110)^b^	5449 (1058)^c^	**0.033**
***Potential moderators***					
SJLsc (min)^3,4^	20 (24)	2 (20)^a^	20 (18)^b^	62 (17)^c^	** < 0.001**
SJLo (min)^3,4^	26 (31)	15 (20)^a^	26 (29)^b^	67 (43)^c^	** < 0.001**
Chronotype (hh:mm)	2:17 (0.40)	1:58 (0.37)^a^	2:16 (0.37)^b^	2:45 (0.51)^c^	** < 0.001**
***Outcome variables***					
BMI z-score^5^	-0.03 (0.96)	0.16 (0.95)	-0.04 (0.94)	-0.10 (1.13)	0.25
Waist to height ratio	0.49 (0.42)	0.50 (0.03)^a^	0.49 (0.03)^b^	0.50 (0.04)^a,b^	**0.004**
***Eating jetlag indicators***					
Energy midpoint jetlag (min)^3,4^	47 (56)	40 (51)	47 (57)	48 (44)	0.44
Eating midpoint jetlag (min)^3,4^	31 (39)	20 (34)^a^	31 (36)^a^	48 (48)^c^	** < 0.001**
First EO jetlag (min)^3,4^	50 (61)	50 (75)^a^	50 (57)^a^	64 (86)^c^	**0.016**
Morning latency jetlag (min)^3,4,6^	36 (44)	34 (40)	36 (43)	36 (42)	0.45
Last EO jetlag (min)^3,4^	25 (37)	15 (45)	25 (3)	30 (53)	0.08
Evening latency jetlag (min)^3,4,7^	36 (44)	34 (40)	36 (43)	36 (42)	0.88

BMI, body mass index; EO, eating occasion; MVPA, moderate to vigorous physical activity; SD, standard deviation; SJLo, social jetlag original.

^1^P-values for continuous variables were derived from one-way ANOVA or the Kruskal–Wallis test for skewed variables, and for categorical variables using the chi-squared test. Bolded values indicate statistical significance (p < 0.05). Tukey’s post hoc test or the Wilcoxon rank-sum test (for skewed variables) was used for pairwise comparisons. Groups that do not share the same superscript letter differ significantly from each other, whereas groups with the same letter do not differ significantly.

^2^Highest parental educational level. Low = high school or vocational diploma or less, medium = associate or bachelor’s degree, high = master’s, licentiate or doctoral degree.

^3^Median (interquartile range, IQR).

^4^Absolute difference.

^5^Based on Finnish references [25].

^6^Total n = 528 (low SJLsc n = 54, moderate n = 424, high n = 50).

^7^Total n = 603 (low SJLsc n = 60, moderate n = 483, high n = 60).

### Association between eating jetlag indicators and adiposity and interaction analysis

None of the eating jetlag indicators were associated with adiposity measures in the full sample (Table 3). However, several statistically significant interactions were observed. SJLsc moderated the associations between eating midpoint jetlag and BMI z-score (P_interact_=0.022), and between morning latency jetlag and both BMI z-score (P_interact_=0.006) and WHtR (P_interact_ <0.001). For SJLo, similar interactions were observed for eating midpoint jetlag on BMI z-score (P_interact_ = 0.039) and morning latency jetlag on WHtR (P_interact_ = 0.011), in addition to an interaction between first eating occasion jetlag and BMI z-score (P_interact_ = 0.002).

Regarding chronotype, interactions were observed between morning latency jetlag and BMI z-score (P_interact_ =0.042), and between evening latency jetlag and WHtR (P_interact_=0.010).

**Table 3:** Associations between eating jetlag indicators and adiposity measures and interactions with social jetlag corrected for sleep debt (SJLsc) and chronotype in the full sample

		**BMI z-score**				**Waist-to-height ratio**			
Eating jetlag variables	n	β	95% CI	P for interaction with SJLsc^1^	P for interaction with chronotype^1^	β	95% CI	P for interaction with SJLsc^1^	P for interaction with chronotype^1^
**Energy intake midpoint jetlag**									
Model 1	639	-0.02	(-0.13, 0.08)			-0.002	(-0.005, 0.002)		
Model 2	617	-0.03	(-0.14, 0.08)	0.99	0.72	-0.002	(-0.005, 0.002)	0.42	0.85
**Eating midpoint jetlag**									
Model 1	639	-0.10	(-0.26, 0.06)			-0.002	(-0.007, 0.003)		
Model 2	617	-0.09	(-0.24, 0.07)	**0.022**	0.61	-0.002	(-0.007, 0.003)	0.73	0.81
**First EO jetlag**									
Model 1	639	-0.07	(-0.17, 0.03)			-0.002	(-0.005, 0.001)		
Model 2	617	-0.06	(-0.16, 0.03)	0.079	0.99	-0.003	(-0.006, 0.001)	0.78	0.23
**Last EO jetlag**									
Model 1	639	0.00	(-0.12, 0.13)			0.001	(-0.002, 0.005)		
Model 2	617	0.03	(-0.09, 0.16)	0.70	0.72	0.002	(-0.002, 0.006)	0.76	0.30
**Morning latency jetlag**									
Model 1	528	0.11	(-0.02, 0.24)			0.004	(-0.000, 0.008)		
Model 2	506	0.11	(-0.01, 0.24)	**0.006**	**0.042**	0.004	(-0.000, 0.008)	** < 0.001**	0.32
**Evening latency jetlag**									
Model 1	603	0.01	(-0.12, 0.14)			0.001	(-0.003, 0.005)		
Model 2	584	0.03	(-0.09, 0.16)	0.38	0.28	0.001	(-0.003, 0.005)	0.15	**0.010**

EO, eating occasion.

Bolded values indicate statistical significance (*p* < 0.05).

^1^Interaction was tested by including an interaction term between the eating jetlag variable and SJLsc/chronotype, both treated as continuous variables, adjusted according to Model 2.

Model 1: Adjusted for age and sex.

Model 2: Model 1 + parental educational level, moderate to vigorous physical activity, sleep duration and total daily energy intake.

### SJL and chronotype stratified analysis

The observed interactions were further examined in stratified analyses.

In SJLsc-stratified analysis, associations were observed only in the high SJLsc group (Table 4). Eating midpoint jetlag was negatively associated with BMI z-score (β:–0.65, 95% CI: − 1.21 to − 0.10, Model 2), whereas morning latency jetlag was positively associated with both BMI z-score (β:0.83, 95% CI: 0.19 to 1.46, Model 2) and WHtR (β: 0.035, 95% CI: 0.013 to 0.057, Model 2).

Similarly, in the SJLo-stratified analyses, associations were observed only in the high SJLo group (Supplemental Table 2). In Model 1, eating midpoint jetlag (β: -0.50, 95% CI: -1.00 to -0.00) and first EO jetlag (β: -0.58, 95% CI: -0.95 to -0.22) were both negatively associated with BMI z-score, while morning latency jetlag was positively associated with WHtR (β:0.029, 95% CI:0.009 to 0.048). In Model 2, these associations attenuated to non-significant, except for the negative association between first EO jetlag and BMI z-score (β:-0.49, 95% CI:-0.84 to -0.14).

**Table 4:** Social jetlag corrected for sleep debt (SJLsc) stratified associations between eating jetlag indicators and adiposity measures

	SJLsc					
	low (10%)		moderate (80%)		high (10%)	
	β	95% CI	β	95% CI	β	95% CI
BMI z-score						
Eating midpoint jetlag						
Model 1	-0.21	(-0.72, 0.30)	0.01	(-0.27, 0.18)	**-0.70**	**(-1.25, -0.15)**
Model 2	-0.19	(-0.70, 0.32)	0.01	(-0.16, 0.18)	**-0.65**	**(-1.21, -0.10)**
Morning latency jetlag						
Model 1	0.13	(-0.19, 0.45)	0.01	(-0.14, 0.15)	**1.04**	**(0.48, 1.60)**
Model 2	0.11	(-0.22 0.44)	0.02	(-0.12, 0.16)	**0.83**	**(0.19, 1.46)**
Waist-to-height ratio						
Morning latency jetlag						
Model 1	-0.006	(-0.003, 0.015)	-0.001	(-0.005, 0.004)	**0.045**	**(0.026, 0.065)**
Model 2	-0.006	(-0.003, 0.015)	-0.001	(-0.005, 0.003)	**0.035**	**(0.013, 0.057)**

Bolded values indicate statistical significance (p < 0.05).

Model 1: Adjusted for age and sex.

Model 2: Model 1 + parental educational level, moderate to vigorous physical activity, sleep duration and total daily energy intake.

In the chronotype-stratified analysis, positive associations were observed among children with evening chronotype tendency in Model 1 for morning latency jetlag and BMI z-score (β: 0.47, 95% CI: 0.00 to 0.94) and for evening latency jetlag and WHtR (β:0.017, 95% CI: 0.002 to 0.032) (Supplemental Table 3). These associations attenuated to non-significant in Model 2.

## Discussion

To the best of our knowledge, this study is the first to examine associations between various eating jetlag indicators and adiposity in preschool-aged children, as well as the potential moderating roles of SJL and chronotype in these associations. We observed no associations between eating jetlag indicators and adiposity measures in the full sample. However, SJLsc was found to moderate these associations. Among children with high SJLsc, greater eating midpoint jetlag (variation in the eating midpoint between weekdays and weekends) was associated with lower BMI z-scores, whereas greater morning latency jetlag (variation in the time between waking and the first EO) was associated with higher BMI z-scores and WHtR. Although chronotype also appeared to moderate some associations, these associations attenuated in chronotype-stratified analysis with further adjustments.

Our findings partly differ from prior research in adults where greater eating midpoint jetlag has generally been associated with higher adiposity [10, 13, 14]. Studies among Spanish/Mexican [10] and Colombian [13] populations have reported positive associations between greater eating midpoint jetlag and higher BMI. The Spanish/Mexican study adjusted for SJL and chronotype, whereas the Colombian did not consider these factors. More recent NHANES findings identified such associations only among evening chronotypes [14]. Our study extends the current evidence to early childhood, incorporating morning and evening latency jetlags as novel indicators, and suggests that associations between eating jetlag and adiposity may only be apparent in the presence of SJL in this age group, as no overall associations were found. Parental regulation of sleep and eating schedules at this age may contribute to the patterns observed, with associations potentially becoming more apparent when sleep timing also fluctuates. However, comparisons with adult studies are not straightforward, as the moderating role of SJL has not been examined previously, and developmental and behavioral differences may further complicate extrapolation to young children. Furthermore, as this is a novel finding emerging only in SJL-stratified analysis, it should be interpreted as exploratory and requires confirmation in future studies.

The moderating role of SJL has not previously been examined in this context and therefore the present findings are exploratory and should be considered hypothesis-generating. Existing literature, however, offers some speculative perspectives that may help to interpret these observations. Circadian regulation of metabolism is influenced by the timing of food intake, as peripheral clocks in metabolic organs respond to feeding cues, as shown in both animal models and human studies [1–4]. For example, an intervention study in adult men found that delaying mealtimes by five hours shifted the phase of metabolic rhythms by delaying the plasma glucose rhythm, without altering sleep [4]. In this context, it is possible that, among children with higher SJL, different indicators of eating timing variability capture distinct aspects of how eating and sleep timing are related. Given that the timing of food intake can influence circadian rhythms, these distinctions may be relevant when considering how eating patterns align or fail to align with shifts in sleep timing. For example, greater eating midpoint jetlag may reflect shifts in eating timing that occur alongside changes in sleep timing, whereas greater morning latency jetlag may relate to a misalignment between waking and eating. This may help explain the contrasting directions of the observed associations. Importantly, these interpretations remain speculative in the context of our study and should be viewed as potential explanations rather than evidence of underlying mechanisms. Replication, preferably in longitudinal is needed before any firm conclusions can be drawn.

We also examined SJL without correction for potential sleep debt (SJLo) for comparison. Overall, the associations observed using SJLo were directionally consistent with those found for SJLsc. However, the associations identified with both SJL definitions (negative association between eating midpoint jetlag and BMI z-score, and positive between morning latency jetlag with both BMI z-score and WHtR) attenuated to non-significant in the fully adjusted SJLo models, suggesting that these associations may be more susceptible to confounding when sleep debt is not accounted for. When using SJLo, we identified one additional association in the high SJLo group: a negative association between first EO jetlag and BMI z-score. These slight variations in the results may suggest that, when sleep debt is not considered, SJL could partially reflect some associations related to sleep debt rather than sleep timing variation alone, but this warrants further investigation. Nevertheless, the overall pattern of results aligns between the SJLsc and SJLo analyses, providing support for our key findings.

Although less consistent, the indications of chronotype interactions in our study are also noteworthy. In adults, associations between eating midpoint jetlag and adiposity appear stronger among evening types [14]. We observed similar patterns among children with an evening chronotype tendency, including positive associations between morning latency jetlag and BMI z-scores and between evening latency jetlag and WHtR, although these associations attenuated in the fully adjusted model. This may reflect the strong association between evening chronotype and high SJL [11], which was also observed in our sample. As SJL more directly captures the misalignment between biological and social time, it may serve as a stronger moderator than chronotype in these associations in children. Further research is needed to clarify the distinct roles of chronotype and SJL in moderating the association between eating jetlag and adiposity.

This study has several strengths and limitations. A key strength is the novel approach, including the use of multiple eating jetlag indicators and the assessment of potential moderating roles of SJL and chronotype in these associations. However, due to the cross-sectional design, no conclusions can be drawn about causality. Another key strength of this study is the use of accelerometer-derived sleep measures, enabling more precise assessment of sleep timing compared to self- or parent-reported data. A relatively large sample size is another strength. However, the participation rate was rather low (24%), and the participating families had a higher than average socioeconomic status [24], which may limit the generalizability of the findings. Furthermore, due to multiple testing in the interaction analyses, we cannot rule out the possibility of type I error. Although the interaction P-values were well below the pre-specified significance threshold and the stratified analyses were consistent with these findings, the exploratory nature of the SJL analyses warrants cautious interpretation and confirmation in future studies. Percentile-based grouping was used to facilitate interpretation; however, this approach is somewhat arbitrary and may have led to a loss of information and a simplified representation. In addition, small extreme groups may have diluted associations and increased the likelihood of chance findings. Furthermore, although chronotype, SJL and some eating jetlag indicators (morning and evening latency jetlag) capture different dimensions of circadian behavior, they are derived from the same sleep timing measures, which may introduce conceptual and mathematical overlap and limit the ability to disentangle their independent effects, The 3-day diet record provided detailed information on eating timing, and collecting food record and accelerometer data on the same days enabled accurate calculation of morning and evening latencies and the derivation of novel morning and evening latency jetlag indicators. SJL indicators and chronotype were derived from sleep data objectively measured using accelerometers. SJL was calculated as the absolute difference between sleep midpoints on workdays and free days using two methods [11, 31], allowing us to explore the potential role of sleep debt. Participants with negative SJL were not analyzed separately, as the primary aim was to study the magnitude of SJL irrespective of its direction [11]. Future studies should consider negative or relative SJL [41], as retaining directionality may reveal additional associations and clarify potential mechanisms. Similarly, while eating jetlag has been operationalised exclusively as an absolute weekend–weekday difference in previous studies [10, 13, 14], applying directional approaches to eating jetlag indicators in future studies could further improve our understanding of these associations. Chronotype was assessed based on the sleep debt-corrected midpoint of sleep on weekend which is a widely used and validated measure [33]. However, as a limitation, we were unable to confirm that children slept without external constraints on weekends, which also applies to the assessment of SJL and eating jetlag. We accounted for a wide range of potential covariates, but residual or unmeasured confounding cannot be ruled out. For instance, we were not able to assess daytime sleep, which may influence the timing of eating and night sleep. Furthermore, because sleep and eating schedules in preschoolers are largely determined by parental practices, the variability observed in these behaviors may reflect parental control oversleep and eating schedules rather than children’s own patterns, potentially reducing our ability to detect overall associations. Finally, our data was collected before the COVID-19 pandemic, and family routines may have changed since then. However, our participants attended preschool, where meal schedules are largely fixed, making it unlikely that these potential changes have affected the associations examined.

In conclusion, we did not observe associations between eating jetlag indicators and adiposity in the full sample. However, our exploratory findings suggest that SJL may moderate the association between eating jetlag and adiposity in young children, as greater variation in eating midpoint was associated with lower BMI, and greater variation in morning latency was associated with higher BMI and WHtR among children with high SJL. These novel findings are hypothesis-generating and as this was the first study to consider the moderating role of SJL in these associations, the findings should be interpreted with caution. Further research is needed to confirm our findings and to better understand the underlying mechanisms.

## Supplementary Information

Below is the link to the electronic supplementary material.

Supplementary Material 1

## Data Availability

The datasets generated during and/or analyzed during the current study are not publicly available due ethical/privacy restrictions but are available from the PI of the DAGIS Survey (ER) via corresponding author upon reasonable request.
