# Supplementary material for: Associations between eating jetlag and adiposity in preschool children, and the moderating roles of social jetlag and chronotype
Source: Eur J Nutr. 2026 Jun 29;65(5):186. doi: 10.1007/s00394-026-04015-6 (PMC13314904; doi:10.1007/s00394-026-04015-6)
Supplement: Supplementary file 1 — Supplementary Material 1 [file 394_2026_4015_MOESM1_ESM.docx]

**Supplementary Information**

**Title:** Associations between eating jetlag and adiposity in preschool children, and the moderating roles of social jetlag and chronotype

**Journal**: European Journal of Nutrition

**Authors**: Mirkka Maukonen^1, 2^, Anna M Ruokolahti^3,4^, Josefine Björkqvist^1,5^, Henna Launistola^1,4^, Jenna Rahkola^1^, Ilse Tillman^1,4^, Henna Vepsäläinen^4^, Carola Ray^1,4^, Eva Roos^1,4,6^, Maijaliisa Erkkola^4^, Reetta Lehto^1^

^1^Folkhälsan Research Center, Helsinki, Finland

^2^ Finnish Institute for Health and Welfare, Helsinki, Finland

^3^University of Tampere, Helsinki, Finland

^4^University of Helsinki, Helsinki, Finland

^5^University of Aberdeen, Aberdeen, United Kingdom

^6^Uppsala University, Uppsala, Sweden

**Corresponding author**: Mirkka Maukonen, mirkka.maukonen@folkhalsan.fi

Supplemental Tables 1-3

| **Supplemental Table 1.** Descriptives of eating jetlag indicators and their source variables in the full sample and by SJLsc groups. Data are presented as mean (SD) or n (%) for categorical variables, unless otherwise indicated. | | | | | |
| --- | --- | --- | --- | --- | --- |
|  |  | SJLsc | | |  |
|  | All n=639 | low n=64 (10%) | moderate n=511 (80%) | high n=64 (10%) | P-value¹ |
| Energy intake midpoint, weekday (hh:mm) | 14:07 (0.46) | 13:59 (0.44) | 14.07 (0.45) | 14:14 (0.48) | 0.14 |
| Energy intake midpoint, weekend (hh:mm) | 14:26 (0.59) | 14:15 (0.56)^a^ | 14:25 (0.58)^a^ | 14.44 (1.05)^c^ | **0.014** |
| Energy midpoint jetlag (relative difference, min) | 19 (66) | 16 (66) | 18 (67) | 30 (64) | 0.38 |
| Energy midpoint jetlag (absolute difference, min)^2^ | 47 (56) | 40 (51) | 47 (57) | 48 (44) | 0.44 |
| Eating midpoint, weekday (hh:mm) | 13:48 (0.30) | 13:43 (0.32)^a^ | 13:48 (0.29) | 13:56 (0.35)^c^ | **0.032** |
| Eating midpoint, weekend (hh:mm) | 14:13 (0.42) | 14:01 (0.39)^a^ | 14:11 (0.41)^a^ | 14:33 (0.44)^c^ | **<0.001** |
| Eating midpoint jetlag (relative difference, min) | 24 (39) | 19 (37)^a^ | 23 (39)^a^ | 37 (42)^c^ | **0.013** |
| Eating midpoint jetlag (absolute difference, min)^2^ | 31 (39) | 20 (34)^a^ | 31 (36)^a^ | 48 (48)^c^ | **<0.001** |
| Wake up time, weekday (hh:mm) | 6:49 (0.31) | 6:39 (0.30)^a^ | 6:50 (0.31)^b^ | 6:51 (0.34) | **0.017** |
| First EO, weekday (hh:mm) | 7:52 (0.37) | 7:46 (0.37) | 7:52 (0.37) | 7:53 (0.38) | 0.45 |
| Morning latency, weekday (min)^3^ | 64 (34) | 68 (35) | 64 (34) | 67 (35) | 0.54 |
| Wake up time, weekend (hh:mm) | 7:20 (0.48) | 7:00 (0.44)^a^ | 7:18 (0.46)^b^ | 7:57 (0.50)^c^ | **<0.001** |
| First EO, weekend (hh:mm) | 8:40 (0.53) | 8:26 (0.51)^a^ | 8:39 (0.53)^a^ | 9:02 (0.52)^c^ | **<0.001** |
| Morning latency, weekend (min)^3^ | 83 (46) | 85 (57) | 85 (45) | 69 (37) | 0.07 |
| First EO jetlag (relative difference, min) | 49 (56) | 40 (57)^a^ | 47 (56)^a^ | 69 (54)^c^ | **0.005** |
| First EO jetlag (absolute difference, min) ^2^ | 50 (61) | 50 (75)^a^ | 50 (57)^a^ | 64 (86)^c^ | **0.016** |
| Morning latency jetlag, (relative difference, min)^3^ | 19 (55) | 17 (68) | 21 (54) | 2 (49) | 0.06 |
| Morning latency jetlag (absolute difference, min)^2,3^ | 36 (44) | 34 (40) | 36 (43) | 36 (42) | 0.45 |
| Last EO, weekday (hh:mm) | 19:45 (0.40) | 19:39 (0.40)^a^ | 19:44 (0.39)^a^ | 19:58 (0.44)^c^ | **0.015** |
| Sleep onset, weekday (hh:mm) | 21:10 (0.38) | 21:04 (0.45) | 21:10 (0.37) | 21:17 (0.43) | 0.16 |
| Evening latency, weekday (min)^4^ | 91 (37) | 89 (36) | 91 (36) | 95 (44) | 0.68 |
| Last EO, weekend (hh:mm) | 19:45 (0.54) | 19:36 (0.49)^a^ | 19:44 (0.53)^a^ | 20:04 (0.59)^c^ | **0.007** |
| Sleep onset, weekend (hh:mm) | 21:29 (0.45) | 21:11 (0.46)^a^ | 21:28 (0.42)^b^ | 21:58 (0.55)^c^ | **<0.001** |
| Evening latency, weekend (min)^4^ | 94 (51) | 89 (52) | 95 (51) | 94 (54) | 0.66 |
| Last EO jetlag (relative difference, min) | 0 (51) | -3 (45) | 0 (52) | 6 (53) | 0.58 |
| Last EO jetlag (absolute difference, min)^2^ | 25 (37) | 15 (45) | 25 (3) | 30 (53) | 0.08 |
| Evening latency jetlag (relative difference, min)^4^ | 3 (54) | 0 (45) | 4 (55) | -1 (51) | 0.72 |
| Evening latency jetlag (absolute difference, min)^2,4^ | 36 (44) | 34 (40) | 36 (43) | 36 (42) | 0.88 |
| Both the relative and absolute values of eating jetlag indicators are presented. Absolute values were used in the analyses to facilitate interpretation and as done in previous studies [10, 13, 14], but they do not directly correspond to the source variables. Relative values are provided for reference. | | | | | |
| SD, standard deviation; SJLsc, social jetlag corrected for sleep debt. | | | | | |
| ¹P-values for continuous variables were derived from one-way ANOVA or the Kruskal–Wallis test for skewed variables, and for categorical variables using the chi-squared test. Bolded values indicate statistical significance (p < 0.05). Tukey’s post hoc test or the Wilcoxon rank-sum test (for skewed variables) was used for pairwise comparisons. Groups that do not share the same superscript letter differ significantly from each other, whereas groups with the same letter do not differ significantly. | | | | | |
| ^2^ Median (interquartile range, IQR). | | | | | |
| ^3^ Total n = 528 (low SJLsc n = 54, moderate n = 424, high n = 50). | | | | | |
| ^4^ Total n = 603 (low SJLsc n = 60, moderate n = 483, high n = 60). | | | | | |

| **Supplemental Table 2**. SJLo stratified associations between eating jetlag indicators and adiposity measures. | | | | | | | |
| --- | --- | --- | --- | --- | --- | --- | --- |
|  | **SJLo** | | | | | |  |
|  | low (10%) | | moderate (80%) | | high (10%) | |  |
|  | β | 95% CI | β | 95% CI | β | 95% CI | P-interact¹ |
| **BMI z-score** |  |  |  |  |  |  |  |
| **Eating midpoint jetlag** |  |  |  |  |  |  |  |
| Model 1 | -0.24 | (-0.88, 0.41) | -0.02 | (-0.20, 0.15) | **-0.50** | **(-1.00, -0.00)** |  |
| Model 2 | -0.17 | (-0.87, 0.53) | -0.01 | (-0.18, 0.16) | -0.36 | (-0.85, 0.13) | **0.039** |
| **First EO jetlag** |  |  |  |  |  |  |  |
| Model 1 | 0.07 | (-0.39, 0.52) | -0.02 | (0.13, 0.08) | **-0.58** | **(-0.95, -0.22)** |  |
| Model 2 | 0.04 | ( -0.45, 0.53) | -0.02 | (-0.12, 0.09) | **-0.49** | **(-0.84, -0.14)** | **0.002** |
| **Waist-to-height ratio** |  |  |  |  |  |  |  |
| **Morning latency jetlag** |  |  |  |  |  |  |  |
| Model 1 | -0.000 | (-0.014, 0.013) | 0.002 | (-0.002, 0.006) | **0.029** | **( 0.009, 0.048)** |  |
| Model 2 | -0.000 | (-0.015, 0.015) | 0.002 | (-0.002, 0.006) | 0.021 | (-0.002, 0.044) | **0.011** |
| EO, eating occasion; SJLo, social jetlag original | | | | | | | |
| Bolded values indicate statistical significance.  ¹ Interaction was tested by including an interaction term between the eating jetlag variable and SJLo, both treated as continuous variables, adjusted according to Model 2. | | | | | | | |
| Model 1: Adjusted for age and sex. | | | | | | | |
| Model 2: Model 1 + parental educational level, moderate to vigorous physical activity, sleep duration and total daily energy intake. | | | | | | | |

| **Supplemental Table 3.** Chronotype stratified associations between eating jetlag indicators and adiposity measures. | | | | | | |
| --- | --- | --- | --- | --- | --- | --- |
|  | **Chronotype** | | | | | |
|  | morning (10%) | | intermediate (80%) | | evening (10%) | |
|  | β | 95% CI | β | 95% CI | β | 95% CI |
| **BMI z-score** |  |  |  |  |  |  |
| **Morning latency jetlag** |  |  |  |  |  |  |
| Model 1 | -0.13 | (-0.59, 0.33) | 0.10 | (-0.05, 0.24) | **0.47** | **(0.00, 0.94)** |
| Model 2 | -0.10 | (-0.62, 0.41) | 0.10 | ( -0.04, 0.24) | 0.25 | (-0.21, 0.72) |
| **Waist-to-height ratio** |  |  |  |  |  |  |
| **Evening latency jetlag** |  |  |  |  |  |  |
| Model 1 | -0.006 | (-0.021, 0.008) | -0.001 | (-0.005, 0.004) | **0.017** | **(0.002, 0.032)** |
| Model 2 | -0.006 | (-0.022, 0.010) | 0.000 | ( -0.004, 0.005) | 0.014 | (-0.001, 0.029) |
| Bolded values indicate statistical significance. | | | | | | |
| Model 1: Adjusted for age and sex. | | | | | | |
| Model 2: Model 1 + parental educational level, moderate to vigorous physical activity, sleep duration and total daily energy intake. | | | | | | |
